# Sera of patients with spontaneous tumour regression and elevated anti‐CA I autoantibodies change the gene expression of ECM proteins

**DOI:** 10.1111/jcmm.13000

**Published:** 2016-10-05

**Authors:** Jan Lakota, Radivojka Vulic, Maria Dubrovcakova, Silvia Tyciakova

**Affiliations:** ^1^ Cancer Research Institute BMC SASBratislavaSlovakia; ^2^ Institute of Normal and Pathological Physiology, SASBratislavaSlovakia; ^3^ St. Elizabeth Cancer InstituteBratislavaSlovakia; ^4^ Institute of Virology BMC SASBratislavaSlovakia

**Keywords:** carbonic anhydrase I, spontaneous tumour regression, basal lamina, anti‐CA I autoantibodies, laminin, collagen

## Abstract

Spontaneous tumour regression after high‐dose therapy and autologous stem cell transplantation is associated with the aplastic anaemia‐like syndrome and the presence of polyclonal autoantibodies against carbonic anhydrase I (CA I). When tumour cells were grown *in vitro* in the presence of patients’ sera positive for anti‐CA I autoantibodies, their morphological pattern was altered. These changes were accompanied by modifications in the gene expression profile. We observed downregulation of genes of the basal lamina assembly (collagen type IV alpha 4, the laminin subunit gamma 2), the extracellular matrix (collagen type I alpha 1), the cytoskeleton (keratin 14 type I), the collagen triple helix repeat containing 1 and the proto‐oncogene WNT7B. On the other hand, the expression of the CA 1 gene was increased in the tumour cells. It was also noticed that the presence of anti‐CA I autoantibodies did not impair tumour cell proliferation and cell viability *in vitro*. These findings were observed only in the presence of patients’ sera positive for anti‐CA I autoantibodies.

## Introduction

Besides the basic research of molecular and cellular mechanisms behind the origin and development of malignant disease, there is a big need to focus on the mechanism of spontaneous tumour regression which can be observed in clinical practice. Spontaneous tumour regression characterizes almost all types of human cancer. The biggest number of cases featuring this phenomenon has been reported in patients with neuroblastoma, renal cell carcinoma, malignant melanoma and lymphoma/leukaemia. The mechanism of spontaneous tumour regression is still unknown, but some of the published data indicates that immune mediation, tumour inhibition by growth factors and/or cytokines, induction of differentiation, hormonal mediation, elimination of a carcinogen, tumour necrosis and/or angiogenesis inhibition, psychological factors, apoptosis and epigenetic mechanisms may be involved in this process 1. An explanation of how and why tumours undergo remission without external treatment would lead to the possibility of improved methods of treating and preventing cancer 2, 3, 4.

High‐dose therapy (HDT) with autologous stem cell transplantation (ASCT) represents one treatment modality for patients with chemosensitive relapsed/refractory malignant diseases. Some of these patients relapsed and developed an aplastic anaemia (AA)‐like syndrome. This AA‐like syndrome is associated with spontaneous tumour regression 5. Nissen and Stern showed that autoimmunity can inhibit the growth of solid tumours and that antitumour activity also promotes autoimmunity against hematopoietic stem cells in acquired AA 6. An interesting fact is that the sera of some of these patients contained autoantibodies against carbonic anhydrase I (anti‐CA I). The presence of these autoantibodies correlated with a significantly increased chance of survival. Carbonic anhydrases (CAs) are zinc‐containing metalloenzymes that catalyse the reversible hydration of carbon dioxide to bicarbonate ion and proton: H_2_O+ CO_2_ ↔HCO_3_^‐^ + H^+^ 7, 8, 9. The CA I enzyme was first purified from bovine erythrocytes 10, but it can also be found in the kidneys, colon, lungs, brain and eyes 11. The CA I is involved in the process of pH homeostasis, respiration and in erythroid differentiation. This protein is also linked with some pathological processes such as anaemia, chronical acidosis, diabetic macular oedema, proliferative diabetic retinopathy and vasogenic oedema 12, 13. Moreover, CA I seems to be a potential biomarker for some cancer types such as colorectal cancer 14, non‐small cell lung cancer 15 and prostate cancer 16. On the other hand, anti‐CA I antibodies were described as important players in some autoimmune diseases such as autoimmune/idiopathic chronic pancreatitis, Sjögren's syndrome 17, idiopathic recurrent pregnancy loss 18, connective tissue diseases 19, systemic lupus erythematosus and other rheumatic diseases. Furthermore, for some autoimmune diseases, anti‐CA I antibodies represent a predictable diagnostic marker 20.

Patients who relapsed after HDT and ASCT and were positive for autoantibodies against CA I, spontaneously regressed with their tumours 5. We used these patients’ sera to examine their effect on biology of tumour cells grown *in vitro*. This study presents the evidence of morphological cell changes and downregulation of the expression of genes associated with basal lamina assembly, cytoskeleton, WNT7B and collagen triple helix repeat containing 1 (CTHRC1) after the incubation of tumour cells with patients’ sera containing high level of anti‐CA I autoantibodies. In contrast, the expression of the CA 1 gene was increased in the tumour cells. These observations may have a possible potential in the design of novel anticancer treatment modalities.

## Materials and methods

### Patients’ samples

Serum samples of a healthy donor and of patients with the AA‐like syndrome and positive for anti‐CA I autoantibodies were obtained at the National Cancer Institute, Bratislava after treatment with HDT (performed according to standard European Bone Marrow Transplantation group protocols) (Table 1). Serum samples were stored at −80°C until analysis. Patients gave written informed consent to the analysis, and the study was approved by the local institutional review board.

**Table 1:** Characteristics of patients' sera used in the study

Patient's ID	Diagnosis	Age (2016)/gender	Transplantation of autologous HSCs	Presence of anti‐CA I autoantibodies	Aplastic anemia‐like syndrome	Morphological changes of tumour cells grown *in vitro* in the presence of sera
SH	Multiple myeloma	44/F	05/2009	+++++	Present	+++++
SK	Multiple myeloma	69/F	01/2003	+++++	Present	+++++
SO	Multiple myeloma	58/F	11/2008	+++++	Present	+++++
SV	Hodgkin's disease	38/M	10/2011	+++	Present	+++
SL	None	56/M	None	(+)	None	(+)
(FS)	(None)	(NA)	(None)	(−)	(None)	(−)

### Cell lines and chemicals

Human breast adenocarcinoma cell line MDA‐MB‐231 (ECACC 92020424), human melanoma cell line A375 (ECACC 88113005), human colorectal adenocarcinoma carcinoma cell line HT29 (ECACC91072201) and human prostate adenocarcinoma cell line derived from metastatic site PC3 (ATCC^®^CRL‐1435TM) were maintained in high‐glucose (4.5 mg/ml) DMEM (Biochrom AG, Berlin, Germany) supplemented with 10% foetal bovine serum (FS) (Biochrom AG), antibiotic/antimycotic mix (10,000 IU/ml penicillin; 5 μg/ml streptomycin; 2.5 μg/ml amphotericin) and 2 mM glutamine. After that, cells were cultivated in 10% human patients’ sera, which were not‐inactivated and/or heat‐inactivated (56°C for 30 min.). All cells were maintained in humidified atmosphere at 37°C and 5% CO_2_. Photographs of live cell cultures were taken at magnification 100× using a light microscope (Carl Zeiss, Jena, Germany).

All chemicals were purchased from Sigma‐Aldrich (St Louis, MO, USA) if not stated otherwise.

### Luminescent cell viability assay

The relative viability of the cells cultivated in different sera was evaluated using CellTiter‐Glo^®^ Luminescent Cell Viability Assay (Promega, Madison, VI, USA). Pentaplicates of cells (6 × 10^2^ in 100 μl media) were seeded in the four or five identical white‐walled 96‐well plates. Measurements were made at 24‐hr intervals after the addition of 100 μl CellTiter‐Glo^®^ Reagent directly to the cultivation media. Luminescence was measured on LUMIstar GALAXY reader (BMG Labtechnologies, Offenburg, Germany) and viability was expressed as a luminescent signal (relative light units ‐ RLU). The RLU produced by the control cells on the last day of the measurement was used to represent 100% cell viability.

### RNA microarray analysis

The RNA microarray analysis was performed using MACS (Miltenyi Biotec GmbH, Bergisch Gladbach, Germany). Total RNA was isolated from triplicates of the breast carcinoma cell line MDA‐MB‐231 cultivated in standard medium with FS (control sample) or with a patient's serum SK (treated sample). The obtained microarray data were background‐corrected and normalized. The clustering and statistical analysis was performed on the normalized data with a reliable signal. Subsequent analysis identified differentially expressed transcripts between the treated sample and the control sample group. Candidate genes with at least 1.5‐fold average downregulated or up‐regulated expression difference were selected and subsequently analysed using the reverse transcriptase quantitative PCR.

### Gene expression analyses

Reverse transcriptase quantitative PCR (RT‐qPCR) was performed using total RNA extracted from 5 × 10^5^ cultured cells using the NucleoSpin RNA II kit (Macherey‐Nagel, Dueren, Germany). RNA was depleted from genomic DNA by DNase treatment (DNase I, RNase‐free; Thermo Fisher Scientific, Waltham, MA, USA) and 1.15 μg of total RNA was reverse transcribed using the SensiFAST cDNA Synthesis kit (Bioline, London, UK). Quantitative RT‐PCR was performed in triplicates in 1× Brilliant III Ultra‐Fast SYBR QPCR Master Mix (Agilent Technologies, Santa Clara, CA, USA), 0.25 pmol/μl concentration of primers and 0.5 μl template cDNA per one reaction. The protocol for RT‐qPCR was started with the activation step at 95°C for 3 min. and followed by 45 cycles of the denaturation step at 95°C for 15 sec. and annealing/polymerization at 60°C for 15 sec. with plate read steps at 75°C and 80°C. PCR was performed in Bio‐Rad 96FX cycler (Bio‐Rad Laboratories, Hercules, CA, USA). The analysis was done using Bio‐Rad CFX Manager software version 1.6 as normalized fold expression using the 2^−ΔΔCt^ method. The genes for hypoxanthine phosphoribosyltransferase 1 (HPRT1) and glyceraldehyde‐3‐phosphate dehydrogenase (GAPDH) were used as the reference genes. The relative decrease or increase in expression in specific mRNA over the control samples was determined. The sequences of primers for selected genes collagen type I alpha 1 (COL1A1), collagen type IV alpha 4 (COL4A4), laminin subunit gamma‐2 (LAMC2), keratin 14 type I (KRT14), CTHRC1, wingless‐type MMTV integration site family member 7B (WNT7B) are shown in Table 2. All oligonucleotides were synthesized by Metabion, Int. (Martinsried, Germany).

**Table 2:** List of primers

Primer	Sequence
COL1A1 for	5′‐GGAATGAAGGGACACAGAGG‐3′
COL1A1 rev	5′‐TAGCACCATCATTTCCACGA‐3′
COL4A4 for	5′‐GGTGCTCCTTCAGATTGACC‐3′
COL4A4 rev	5′‐GACCCCTTTTCAGGAACACA‐3′
LAMC2 for	5′‐GGATTCAGTGTCTCGGCTTC‐3′
LAMC2 rev	5′‐TGCTGTGCTTCTTCTTTCCA‐3′
KRT14 for	5′‐CGAGATGCGTGACCAGTATG‐3′
KRT14 rev	5′‐TGTTCTCCAGGGATGCTTTC‐3′
CTHRC1 for	5′‐GCTCACTTCGGCTAAAATGC‐3′
CTHRC1 rev	5′‐CAGCACCAATTCCTTCACAA‐3′
WNT7B for	5′‐GTCCTGTACGTGAAGCTCGG‐3′
WNT7B rev	5′‐GTACTGGCACTCGTTGATGC‐3′
CA1 for	5′‐GCAAGTCCAGACTGGGGATA‐3′
CA1 rev	5′‐TCAGCACTGATCGGTTATCG‐3′
GAPDH for	5′‐GAAGGTGAAGGTCGGAGTC‐3′
GAPDH rev	5′‐GAAGATGGTGATGGGATTTC‐3′
HPRT1 for	5′‐GACCAGTCAACAGGGGACAT‐3′
HPRT1 rev	5′‐CCTGACCAAGGAAAGCAAAG‐3′

## Results

### Clinical and laboratory characteristics of patients with spontaneous tumour regression

Spontaneous tumour regression of after HDT and ASCT is associated with AA‐like syndrome and the presence of high or moderate levels of polyclonal autoantibodies against CA I 21. The serum marked as ‘SK’ was obtained from a 68‐year‐old female patient of Central European origin with a past medical history including multiple myeloma diagnosed in January 2002 (Table 1). After the diagnosis, she underwent standard chemotherapy – four cycles of VAD (vincristine, doxorubicin and dexamethasone) without significant response to chemotherapy. The autologous stem cells were collected after priming with cyclophosphamide (2 g/m^2^) and the CD34^+^ cells were positively selected. In January 2003 she was treated by HDT (melphalan 200 mg/m^2^) with ASCT. The other patients (SO, SH) had a very similar medical record but one patient (SV) was with Hodgkin's disease (Table 1). After HDT and ASCT, all patients relapsed/progressed with their disease and achieved a spontaneous regression without any further treatment. All tested patients’ sera were confirmed to be anti‐CA I positive at concentration of 0.2% (1:500 dilution) using Western blot (WB) analysis.

### The presence of anti‐CA I autoantibodies changes tumour cell morphology *in vitro* and changes the gene expression profile

**Figure 1:**
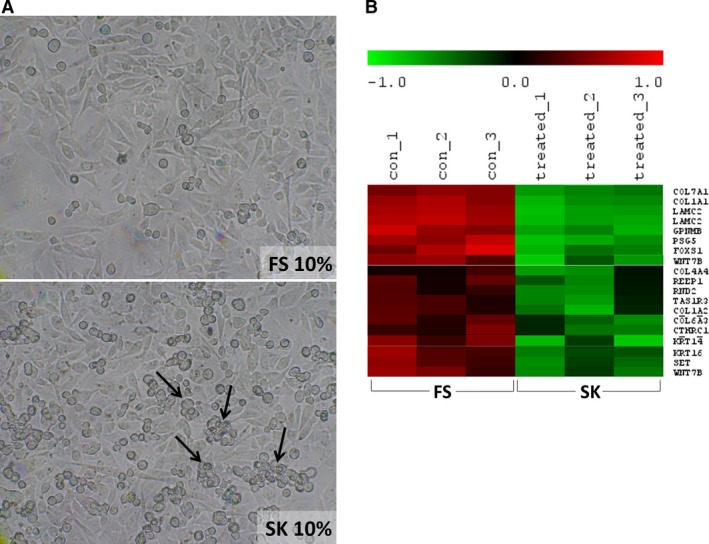
Morphological and expression profile changes of cultures of MDA‐MB‐231 breast carcinoma cells in the presence of a patient's serum with a high level of autoantibodies against carbonic anhydrase I and with an aplastic anemia‐like syndrome. (**A**) A light‐microscope image of living cells with changed morphology cultivated in patient's serum (SK) on day 7 compared to cells cultivated in a medium containing foetal serum (FS) negative for anti‐CA I autoantibodies, magnification 100×. (**B**) Statistically significantly downregulated genes associated for example with adhesion, cytoskeleton and a proto‐oncogene WNT7B; RNA microarray map.

To reveal possible molecular mechanisms of spontaneous regression of tumours, we studied the effect of anti‐CA I positive‐patients’ sera on the growth of different tumour cell lines *in vitro*. First, we focused on the effect of 10% anti‐CA I positive patient serum (SK) on the growth of MDA‐MB‐231 breast carcinoma cells compared to the effect of 10% constitutionally negative FS. Tumour cells grown in the presence of the SK positive patient serum developed the following morphological changes: they transformed into round cells with a tendency to clump together, detaching from the bottom of the Petri dish, and forming spheroids (Fig. 1A). It is important to notice that we observed the same morphological changes of the tumour cells after treatment with heat inactivated (56°C for 30 min.) or heat non‐inactivated serum (data not shown). Therefore, we continued to work with the heat non‐inactivated serum to be closer to the physiological status.

Subsequently, we decided to perform RNA microarray analysis to more closely study the cause of these morphological changes. The results were in agreement with the observed morphological findings and revealed changes in the expression profile as stated in the RNA microarray report: ‘The downregulated genes in treated (SK) compared to untreated (FS) samples include several genes related to adhesion and cytoskeleton, such as different collagens and keratin family members. In addition, some proto‐oncogenes WNT7B and GLI2 are downregulated too’ (Fig. 1B).

### Patients’ sera containing anti‐CA I autoantibodies induce similar morphological changes of tumour cell lines of four different origins

Obtaining the data from the RNA microarray analysis, we focused our attention on RT‐qPCR expression analysis of some pronounced downregulated genes of the basal lamina assembly (COL4A4, LAMC2), extracellular matrix (COL1A1), cytoskeleton (KRT14) and proto‐oncogene WNT7B and CTHRC1 (Table 1). After a long search, we finally obtained bona fide anti‐CA I negative control human serum (negative on WB at 1:50 dilution) from a healthy donor (SL) and thus acquired a more accurate control for further experiments. The morphological pattern of the tumour cells grown in the presence of this negative human serum did not show significant changes in comparison with morphological pattern of the tumour cells cultivated in the presence of the foetal serum.

**Figure 2:**
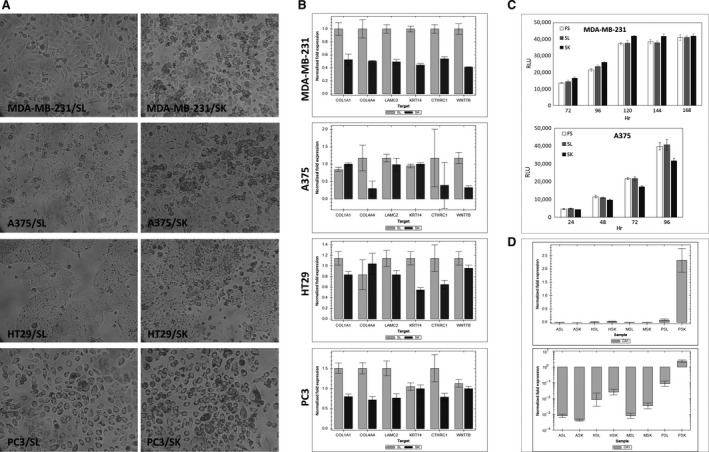
Cell lines from four different sources show changes in the presence of patient's serum (SK) with a high level of anti‐CA I autoantibodies. (**A**) Cells with changed morphology after cultivation in patient's serum (SK) compared to cells cultivated in a control human serum (SL) negative for anti‐CA I autoantibodies; light‐microscope picture, magnification 100×. (**B**) Selected genes responsible for basal lamina assembly (COL1A1 – alpha‐1 type I collagen, COL4A4 – alpha‐4 type IV collagen, LAMC2 – laminin subunit gamma‐2, KRT14 – keratin 14 type I, CTHRC1 – collagen triple helix repeat containing 1, WNT7B – Wingless‐Type MMTV Integration Site Family, Member 7B) are downregulated in cells cultivated in patient's serum (SK); reverse transcriptase quantitative PCR (RT‐qPCR). (**C**) The viability and growth of cells cultivated in patient's serum SK is not impaired; luminescent cell viability assay detecting intracellular ATP. (**D**) The expression of cellular CA I is up‐regulated in the presence of anti‐CA I autoantibodies; RT‐qPCR; ASL, ASK – A375 melanoma cells cultivated in SL or SK serum; HSL, HSK – HT29 colon carcinoma cells in SL or SK serum; MSL, MSK – MDA‐MB‐231 breast carcinoma cells in SL or SK serum; PSL, PSK – PC3 prostate cancer cells in SL or SK serum.

Subsequently, we compared the morphology of the different cell lines MDA‐MB‐231 (breast carcinoma), HT29 (colon carcinoma), A375 (malignant melanoma), PC3 (prostatic carcinoma) cultivated in the presence of 10% SK positive patient's serum and in the presence of 10% control human serum (SL). Tumour cells grown in the presence of the positive patient's serum developed the same morphological changes as described above (Fig. 2A). The results from the RT‐qPCR confirmed downregulated expression of the mRNAs for all selected genes (Fig. 2B). Nevertheless, we did not observe any differences between the levels of expression of the selected genes in the cells cultivated with control serum SL compared to the levels of the same genes in the cells cultivated with standard medium containing FS. Downregulated mRNA levels for proteins of the basal lamina and cytoskeleton were in agreement with the observed morphological changes.

### Anti‐CA I autoantibodies do not impair tumour cell viability and proliferation

The luminescent cell viability assay detecting intracellular ATP of viable cells has been performed in MDA‐MB‐231 and A375 cell lines cultivated in 10% FS, SL and SK sera during five and four consecutive days, respectively (Fig. 2C). This test clearly reflects practically no difference in the viability and proliferation of the cells treated in the presence of foetal serum, negative and positive human serum.

### The presence of anti‐CA I autoantibodies increases the expression of CA I protein in tumour cells *in vitro*

To analyse the effect of anti‐ CA I autoantibodies on CA I expression, we cultivated tumour cells with patient's serum (SK) containing high titres of anti‐CA I autoantibodies (Table 1). The mRNA for CA 1 was found to be expressed differently in different cell lines (PC3>HT29>MDA231>A375). However, the treatment with anti‐CA I positive serum increased the level of CA 1 mRNA in PC3 and HT29 treated cell lines (Fig. 2D). In the cell lines MDA‐MB‐231 and A375 we observed a small increase in CA 1 expression, but the expression of CA 1 mRNA was only slightly above the background.

### Four different patients’ sera with spontaneous tumour regression have similar effects on the morphology and gene expression of tumour cells

**Figure 3:**
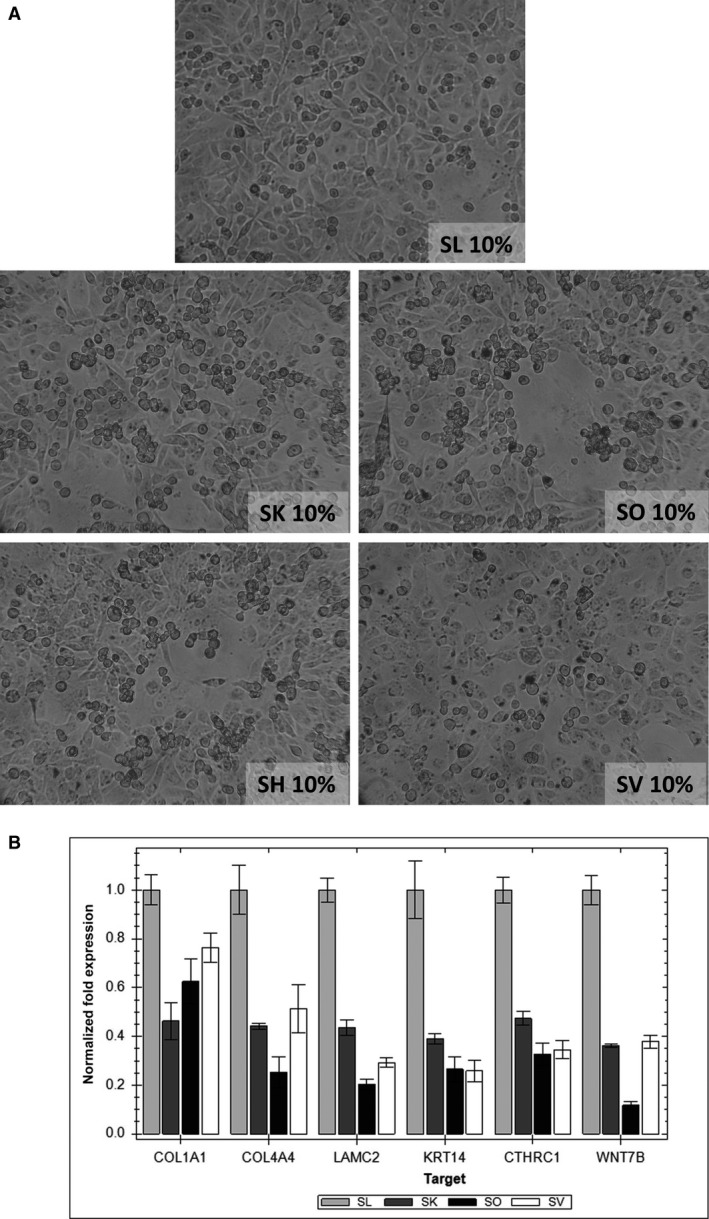
Morphological changes of cultures of MDA‐MB‐231 breast carcinoma cells in the presence of four patient's sera with a high level of autoantibodies against carbonic anhydrase I. (**A**) A light‐microscope picture of living cells with changed morphology cultivated in patients’ sera: SK, SO, SH, SV and in a control human serum (SL) from a healthy donor negative for anti‐CA I autoantibodies, magnification 100×. (**B**) Selected genes responsible for adhesion, cytoskeleton and WNT signalling (COL1A1 – alpha‐1 type I collagen, COL4A4 – alpha‐4 type IV collagen, LAMC2 – laminin subunit gamma‐2, KRT14 – keratin 14 type I, CTHRC1 – collagen triple helix repeat containing 1, WNT7B – Wingless‐Type MMTV Integration Site Family, Member 7B) are downregulated in cells cultivated in patients’ sera; reverse transcriptase quantitative PCR.

The MDA‐MB‐231 cells were cultivated in the presence of 10% sera from different patients (SH, SO, SV) and a control serum from healthy donor (SL). When the cells were grown in the presence of these positive patients’ sera we observed the same morphological changes (detachment from the bottom of the Petri dish, clumping, transformation into round cells and spheroids) as in the case of the positive serum (SK) (Fig. 3A). The results from the RT‐qPCR showed the downregulation of the mRNAs coding the proteins of the basal lamina (collagen IV and laminin gamma), extracellular matrix (collagen I), cytoskeleton (cytokeratin 14), proto‐oncogene WNT7B and CTHRC1 (Fig. 3B).

## Discussion

Spontaneous tumour regression is manifested as the complete or partial disappearance of a malignant tumour without treatment or in the presence of therapy that is considered inadequate in exerting a significant influence on neoplastic disease. Renal cell carcinoma, malignant melanoma, neuroblastoma, leukaemia and non‐Hodgkin's lymphoma are some of the cancer types that can undergo spontaneous regression 1, 22. Various mechanisms have been proposed to explain the mechanism of spontaneous regression of tumours, such as immunologic factors, concomitant infections, hormonal factors and genetic factors, elimination of carcinogens, surgical trauma of the primary tumour and induction of differentiation. Of these, it seems that immunological factors are strongly associated with this phenomenon 3, 23.

In previous work, we have reported that some patients with Hodgkin's disease and multiple myeloma relapsed after HDT with ASCT 5. Interestingly, we have found that sera of these patients contained high titres of autoantibodies against carbonic anhydrase isoform I (CA I). The presence of CA I autoantibodies in patients’ sera indicated an improved prognosis.

At the beginning, we focused on the effect of 10% anti‐CA I positive patient serum (SK) on the growth of MDA‐MB‐231 breast carcinoma cells compared to the effect of 10% constitutionally anti‐CA I negative foetal bovine serum ‐ marked as FS (*i.e*. standard cultivation conditions). We have observed impaired cellular morphology, including the rounding of cells, the formation of spheroids, clumping and detaching of cells from the bottom of the Petri dish. RNA microarray analysis showed that this effect is probably caused by the decreased expression of several cytoskeletal genes and genes involved in the process of cell adhesion and the formation of extracellular protein structures, such as different collagens and keratin family members. Moreover, some proto‐oncogenes were downregulated in the SK‐treated MDA‐MB‐231 cells as well. These included WNT7B and GLI2 genes.

On the other hand, we have observed that sera from healthy donors are ‘negative’ using WB analysis at 1:500 dilution. However, at a higher concentration (1:50 dilution), a positive band indicating anti‐CA I autoantibodies is almost always present also in the sera of the healthy population (unpublished observation). Later, we were able to find an anti‐CA I bona fide human negative serum of a healthy donor (*i.e*. negative on WB at 1:50 dilution) which we have used in further experiments as a proper control.

Therefore, we have focused on the downregulated genes of the MDA‐MB‐231 breast carcinoma cell line grown in the presence in the positive (SK) and negative (SL) human serum. We focused on some pronounced downregulated genes of the basal lamina assembly (COL4A4, LAMC2), extracellular matrix (COL1A1), cytoskeleton (KRT14) and proto‐oncogene WNT7B and CTHRC1. In contrast with the growth in the presence of the negative human serum (SL), MDA‐MB‐231 cells grown in the presence of the anti‐CA I positive human serum (SK) exhibited the same abovementioned morphological changes. Moreover, RT‐qPCR analyses showed that mRNAs of the genes COL4A4, LAMC2, COL1A1, KRT14, WNT7B and CTHRC1 were downregulated. The same results were obtained when the MDA‐MB‐231 breast carcinoma cells were grown in the presence of three sera from different patients (SO, SH, SV) with a high level of autoantibodies against CA I and a spontaneous regression of their malignancy.

Furthermore, we focused on three other cell lines of different origins – A375 (malignant melanoma), HT29 (colon carcinoma) and PC3 (prostatic carcinoma) – where we observed a very similar pattern of morphological changes and downregulation of the genes COL4A4, LAMC2, CTHRC1 and proto‐oncogene WNT7B when the cells were grown in the presence of the anti‐CA I positive serum (SK).

Both type IV collagen and LAMC2 play a key role in establishing the basal lamina. In the present study, we showed that the mRNA levels of these two proteins were downregulated approximately to one‐half after cultivation in the presence of 10% patients’ sera positive for autoantibodies against CA I. The cellular morphological pattern was clearly in agreement with the data obtained by methods of molecular biology. Type IV collagen is one of the most dominant components of the basement membrane which defines the tumour microenvironment and provides significant host‐derived regulatory signals during the progression of tumour growth and metastasis 24. It was found that type IV collagen supports the adhesion and spreading of many different cell types such as endothelial, breast cancer cells, pancreatic cancer cells, myeloma, fibrosarcoma, glioma and neuroblastoma. Type IV collagen also plays an important role in the processes of angiogenesis, tissue remodelling and cancer progression. Very comparable data were reported for the other epithelial basement membrane protein LAMC2. The elevated expression of LAMC2 in tumour cells appears to drive tumourigenesis through its interactions with several cell‐surface receptors including α6β4 and α3β1 integrins and epidermal growth factor receptors. Published data indicate that the LAMC2‐mediated signalling network plays an important role in the progression, migration and invasion of multiple types of cancer, suggesting that it might be a potential therapeutic anticancer target for inhibiting tumourigenesis 25. It was found that elevated LAMC2 increased traction force, migration and invasion of colon cancer cells and lung adenocarcinoma cells accompanied by the induction of epithelial‐mesenchymal transition 26, 27.

The WNT7B gene may play important roles in the development and progression of gastric cancer, oesophageal cancer and pancreatic cancer. It was found that during the early progression of metastasis, the WNT7B signalling pathway is involved in the migration and invasion of cancer cells 28. This process is known as the epithelial‐mesenchymal transition and leads to the transformation of epithelial cells into mesenchymal cells. These changes are likely to be involved in cancer development and progression 29. Moreover, the Wnt7B is a characteristic oncogenic marker of the triple negative breast carcinoma cell line MDA‐MB‐231 30. The downregulation of the mRNA coding the WNT7B gene caused by the anti‐CA I positive human serum can directly implicate a possible antitumour effect.

On the other hand, it has been found that expression of CTHRC1 is up‐regulated in several malignant tumours such as melanoma, and cancers of the gastrointestinal tract, breast, thyroid, liver and pancreas 31. CTHRC1 can stabilize the physical interaction between Wnt ligands and Frizzled receptors, and selectively activate the non‐canonical Wnt pathway to regulate cell motility and taxis 32. Our results showed that the mRNA levels of the CTHRC1 gene were decreased after treatment with sera from patients with spontaneous regression of malignant disease with high titres of anti‐CA I autoantibodies in all four tested tumour cell lines. This further suggests a possible antitumour effect of the sera from patients with spontaneous tumour regression.

An interesting experimental finding is that the CA 1 mRNA levels were up‐regulated in all four different tumour cell lines if these cells were cultivated in the presence of a positive serum. This experiment clearly shows different base lines of CA I expression in different cell lines and an up‐regulation of the CA 1 mRNA, although it was not statistically significant in two of the four lines. We do not have an explanation for this observation; however, it seems the cells are ‘trying’ to enhance the levels of the CA I enzyme.

On the other hand, the decreased levels of mRNAs encoding type I collagen, type IV collagen, laminin subunit gamma 2 and two proto oncogenes WNT7B and CTHRC1, suggest a complex and profound effect of the sera of patients with spontaneous tumour regression on the cancer cells. It seems that these mRNAs are simultaneously downregulated. Finally, observed morphological changes of the cells are probably because of the ‘synergistic’ effect of this concomitant mRNA downregulation. In all studied sera, this effect is accompanied by the presence of high titres of autoantibodies against CA I. It is tempting to speculate how the observed effects on different tumour cell lines *in vitro* can explain the spontaneous tumour regression in the patients. This spontaneous tumour regression is always accompanied by AA‐like syndrome 5, 6. From pathophysiological point of view the malignant and haemopoetic cells are probably not able to attach to and to synthetize the basal lamina and thus not able to perform further ‘tissue’ functions *i.e*. tumour growth and normal haemopoesis. The CA I is an ‘obscure’ enzyme. The presented data suggest its possible central role in the tissue growth, nevertheless, with a mechanism which needs to be established.

## Conflict of interest

The authors confirm that there are no conflicts of interest.
